# The effect of diabetes on mortality of COVID-19: A protocol for systematic review and meta-analysis

**DOI:** 10.1097/MD.0000000000020913

**Published:** 2020-07-02

**Authors:** Yan Yang, Wen Zhong, Yuan Tian, Chunguang Xie, Xiaoxu Fu, Hui Zhou

**Affiliations:** a Chengdu University of traditional Chinese medicine; b Affiliated Hospital of Chengdu University of traditional Chinese Medicine, Sichuan, China.

**Keywords:** COVID-19, diabetes, meta-analysis, mortality, protocol, systematic review

## Abstract

**Background:**

Novel coronavirus pneumonia (COVID-19) is a very serious and urgent infectious disease. With the development of global economy and the improvement of living standard, the incidence of diabetes is increasing year by year. And it is more common in the elderly. COVID-19 is associated with much chronic disease, especially diabetes. At present, there is no systematic review and meta-analysis of mortality based on large scale of data between diabetes and COVID-19 all over the world.

**Methods and analysis:**

The databases of PubMed, the Cochrane Library, EMBASE, Wanfang Data, China National Knowledge Infrastructure database (CNKI) and VIP were searched by computer, and the researches related to diabetes mellitus and mortality of COVID-19 were collected. The searching time was from the establishment of the database to April 30 2020. The meta-analysis was carried out by Review Manager Version 5.3 and stata 14.0 software for Mac software after 2 researchers independently selected literature, extracted data and evaluated the bias risk. The main outcome was the mortality of COVID-19 which was included in meta-analysis and subgroup analysis. The bias of the study was evaluated independently by NOS scale, and published by funnel chart. The sensitivity was analyzed row by row.

**Results:**

The results will be published at a peer-reviewed journal.

Registration number: INPLASY202040158.

## 1 Introduction

Diabetes mellitus (DM) is a complex chronic disease characterized by high blood glucose, absolute and relative insulin deficiency. It includes type1 diabetes, type 2 diabetes and special type diabetes. Among them, the morbidity of type 2 diabetes was the highest. The global prevalence of T2 DM in 2017 is estimated to be 4.63 billion.^[1]^ In 2017, the total number of deaths caused by high fasting plasma at all ages was 6.5 million, of which type 2 diabetes accounted for 1 million deaths.^[2]^ Novel coronavirus pneumonia (COVID-19) caused by severe acute respiratory syndrome coronavirus 2 (SARS-CoV-2) is seriously endangering human life and health and property safety. March 11, 2020 by the World Health Organization (who) announced as a global pandemic.^[3]^ Many early studies have found that patients with chronic diseases such as diabetes are more severe and have worse prognosis.^[4–8]^ A number of retrospective analysis of patients with COVID-19 showed that the prevalence of diabetes patients increased in varying degrees, and the condition was usually more serious than that of general patients.^[9–11]^ The analysis in China by Chinese Center for Disease Control and Prevention (CDC) shows that the mortality combined with diabetic patients is 7.3%, while the overall mortality is 2.3%.^[12]^ The National Institutes of health in Italy reported that the prevalence of diabetes in patients who died of SARS-CoV-2infection was 35.5%.^[13]^ According to the preliminary data from the American Centers for Disease Control and Prevention on March 28, 2020, diabetes is the most common basic health condition among the people infected with SARS-CoV-2, about 10.9%. Furthermore, it is estimated that 32% of the patients who need to be admitted to ICU.^[14]^ Diabetic patients are more likely to suffer from serious infection due to hyperglycemia, chronic inflammatory state, microcirculation damage and other factors.^[15]^ It was found that type 2 diabetes may increase the expression of Angiotensin Converting Enzyme 2 (ACE2) in the lung.^[16]^ The novel coronavirus pneumonia is promoted by ACE2 as a binding site of SARS-CoV-2, but the decrease of ACE2 expression may result in severe lung injury after infection.^[17]^ Serious infections, serious diseases and glucocorticoids can damage insulin sensitivity, so infectious diseases lead to high mortality of diabetic patients.^[18]^ However COVID-19 mortality combined with diabetes is still not clear. The duration, age, gender, race and blood glucose control of diabetes may have effect on the mortality of COVID-19. Our study will solve that by meta-analysis and it is very necessary.

## 2 Methods and analysis

### 2.1 Study registration

The report of this system review plan is in accordance with the preferred report item of the system review and meta-analysis plan (PRISMA-P) guidelines.^[19–20]^ This study will be conducted in accordance with the PRISMA extension statement of NMA.^[21]^

### 2.2 Inclusion and exclusion criteria

Population: adult dead from COVID-19 will be included in our study. There are no restrictions on the region, gender and age of patients.

Intervention: This study will investigate comparisons of diabetes and non-diabetes COVID-19 patients. According to whether diabetes mellitus is combined, they are divided into diabetes group (trail) and no diabetes group (comparison). Patients who do not die will be excluded.

Outcomes: the primary outcome will be the mortality ofCOVID-19 patients. Secondary outcomes will include the blood glucose control level and inflammatory markers.

Study design: All studies on mortality of COVID-19 will be included in this study.

### 2.3 Outcomes

The mortality of COVID-19

FBG (fasting blood glucose)

PBG (postprandial blood glucose)

HbA1c (glycated hemoglobin)

CRP (C-reactive protein)

### 2.4 Study search

Computer retrieval three English database including PubMed, EMBASE, the Cochrane Library, and 4 Chinese databases including China National Knowledge Infrastructure (CNKI) database, Wanfang Data Knowledge Service Platform, the VIP information resource integration service platform (cqvip), China Biology Medicine Disc (Sino Med) will be searched from their inception to April 30 2020, with a language limitation of English and Chinese. In addition, Google scholar and Baidu Scholar will be used to find out potential missing papers. There is no time limitation about literatures. Novel coronavirus pneumonia, COVID-19, mortality, prognosis, and blood glucose control, diabetes, type 1 diabetes, type 2 diabetes, new coronavirus pneumonia, CRP, are the key words for search. To identify other eligible studies, reference lists of relevant publications will be reviewed for a manual search. An example of search process is presented in Table 1.

**Table 1:**
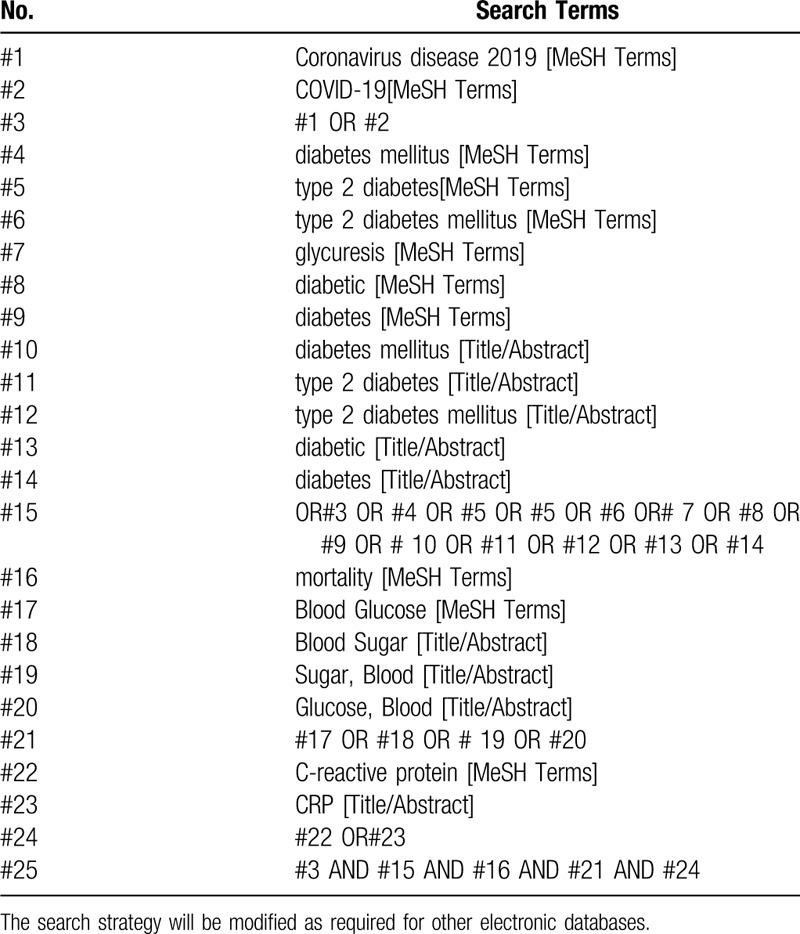
Search strategy for PubMed.

### 2.5 Study selection

**Figure 1:**
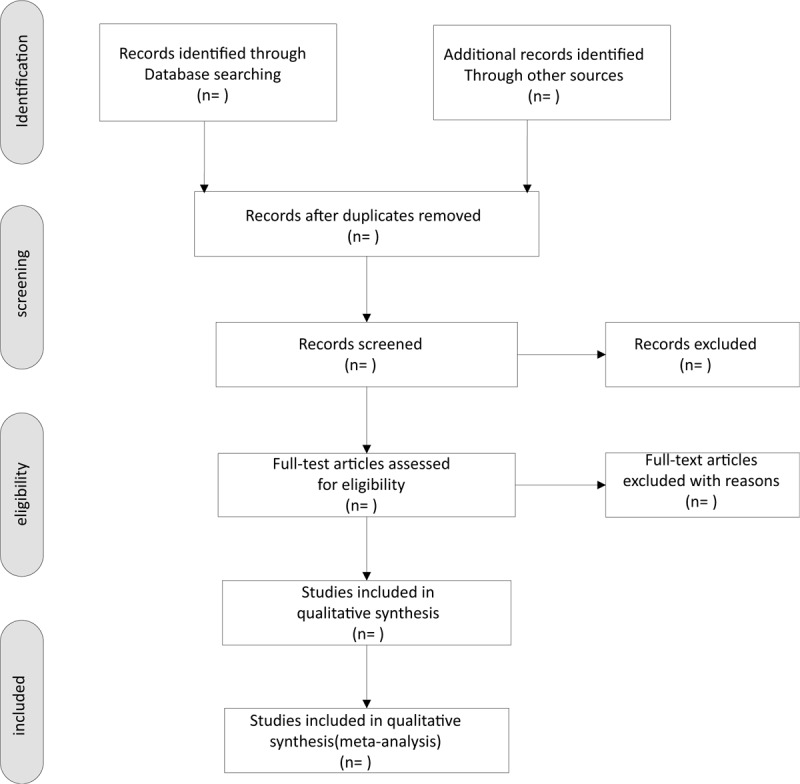
Flow chart of the study selection.

Based on the pre-determined inclusion criteria, two independent reviewers will evaluate all titles and abstracts to exclude papers that are not considered relevant. The remaining provisions will be included in a further assessment. Reviewers will carefully examine the full text of each potentially relevant article. The process of study identification and exclusion will be described by PRISMA flow chart. Differences in research options will be resolved through consultation, and record in Excel file (Fig. 1.).

### 2.6 Data extraction and exclusion

Two researchers independently screened literature, extracted data and cross checked them. In case of any difference, it shall be settled through discussion or consultation with a third party. In the process of literature selection, the first step is to read the title. After excluding the obviously irrelevant literature, the second step is to read the abstract and the full text to determine whether to include it. If necessary, contact the author of the original study by email or phone to obtain the uncertain but very important information for this study. The content of data extraction includes:

1. Basic information: the first author, publication time, research location, sample size, sex ratio, age, research type;
2. Outcome indicators of concern;
3. Relevant elements of bias risk assessment.

#### 2.6.1 Exclusion criteria

1. Exclude non-Chinese and English articles;
2. Literature with repeated reports;
3. Literature without relevant outcome indicators;
4. Literature with incomplete or missing analysis data and unable to contact the original author.

### 2.7 Risk of bias assessment

Bias risk assessment included in the study: 2 researchers independently assessed the bias risk of the included study with NOS scale, and cross checked the results.

### 2.8 Data analysis

Data analysis will be conducted in Review Manager Version 5.3 and stata 14.0 software for Mac. The risk ratio (RR) was used as the analysis statistic and 95% CI was provided. The heterogeneity of the results was analyzed by χ^2^ test (the test level was α = 0.1), and the degree of heterogeneity was determined byI^2^. If there is no statistical heterogeneity between the results of each study, the fixed effect model is used for meta-analysis; if there is statistical heterogeneity between the results of each study, the source of heterogeneity is further analyzed. After excluding the influence of obvious clinical heterogeneity, the random effect model is used for meta-analysis. The level of meta-analysis is set as α = 0.05. Significant clinical heterogeneity was treated by subgroup analysis or sensitivity analysis, or only descriptive analysis.

### 2.9 Investigation of heterogeneity

If there is substantial heterogeneity between studies, then we will conduct subgroup analysis to explore the heterogeneity. To avoid post hoc analysis, the subgroup analysis will be conducted according to 3 hypotheses: race, course of diabetes, glucose level. To further improve the reliability of subgroup analysis, we will evaluate the credibility of our subgroup analysis according to the guidance for credible subgroup analysis. If there are enough studies included, then meta-regression will be conducted to further explore the heterogeneity. Those subgroup effects that occur simultaneously in subgroup analysis and regression analysis will be considered credible.

### 2.10 Sensitivity analysis

Draw funnel chart for all-cause mortality outcome indicators of diabetes and no diabetes COVID-19 patients. To ensure the stability of the results, we will conduct sensitivity analysis of the results by excluding each of the studies included in the analysis one by one, then re-analyzing the results, and comparing the differences between the re-obtained results and the original results. In this way, we will be able to assess the impact of individual studies on overall outcomes and their robustness.

### 2.11 Reporting bias assessment

The integrity of the studies is an important factor affecting the accuracy of the results and conclusions of meta-analysis. The integrity of the included studies is mainly measured by reporting bias, of which publication bias is the most common. Therefore, this study will identify report bias by publication bias assessment. A funnel plot will be drawn to investigate the publication bias. Funnel plot will be asymmetric when publication bias exists, such as when research with small sample and no statistically significant results are not published. The more obvious the asymmetry of funnel plot is, the more likely there is publication bias^[22]^ And then Egger test will be conducted for statistical assessment the publication bias. The publication bias is considered to exist if *P* < .05^.^^[23]^

### 2.12 Patient and public involvement

No patients or public will participate in the study.

### 2.13 Ethics and dissemination

Since confidential patient data will not be involved in this study, formal ethics approval is not required. The frame- work of the PRISMA statements for NMA will be applied to guide review authors to perform this study. The results will be disseminated by a peer-reviewed publication.

## 3 Discussion

COVID-19 is widespread all over the world, causing great casualties and property losses. It is a battle related to all mankind. This study is a comprehensive and systematic review, aim to compare the mortality of COVID-19 between diabetes and non-diabetes. We will analyze the sex, age, course of disease, race and blood glucose control level of COVID-19 dead patients. So that the prognosis of patients with new diabetes mellitus will be predicted and the blood glucose control should be strengthened in the clinical treatment. The epidemiology of COVID-19 incidence, severity of illness and mortality seem to be shifted towards older people particularly those with diabetes, hypertension, and cardiovascular disease.^[24]^ Considering research costs and data arrange our study did not analyze diabetes related complications. Next study will consider providing a systematic review of the role of diabetes complications in the development of COVID-19. Meanwhile this is of great practical significance to clarify the harm of diabetes to public health. It can also provide reference for clinicians.

### 3.1 Amendments

If any modification is required, we will update our protocol to include any changes in the entire research process.

## Author contributions

**Conceptualization:** Yan Yang, Wen Zhong, Chunguang Xie.

**Data curation:** XiaoXu Fu.

**Formal analysis:** Yan Yang, Wen Zhong.

**Funding acquisition:** Chunguang Xie.

**Investigation:** Yuan Tian.

**Methodology:** Yan Yang, Wen Zhong, Chunguang Xie.

**Project administration:** Chunguang Xie.

**Resources:** Yan Yang, Chunguang Xie.

**Software:** Yan Yang, Wen Zhong.

**Supervision:** XiaoXu Fu.

**Writing – original draft:** Yan Yang.

**Writing – review & editing:** Chunguang Xie, Hui Zhou.
